# Supplementary material for: Successes and challenges of an online based nutrition awareness program in 9–11-year-old children in four Arab countries: The Ajyal Salima digital platform qualitative study
Source: PLoS One. 2026 Mar 11;21(3):e0325583. doi: 10.1371/journal.pone.0325583 (PMC12978466; doi:10.1371/journal.pone.0325583)
Supplement: S2 Table — (DOCX) [file pone.0325583.s002.docx]

| **Table 2 – Quotes from Parents, Teachers, and Students Categorized by Themes and Sub-Themes  Thematic Analysis of Quotes from Parents, Teachers, and Students** | | |
| --- | --- | --- |
| **Theme** | **Description** | **Sub-Themes** |
| **1. Usability and Support Using the Digital Platform** | Focuses on the ease of use, challenges, and support systems available for students, parents, and teachers. | **1.1** **Technical Challenges and Registration Issues**  **Palestine:**  **Parents:**   - At first, we faced a problem with the email address where it would get mixed up when we wrote it down, but my husband fixed it for us. - Opening the link and entering the platform were challenging.   **Teachers:**   - They reported a problem where the platform would stop for a while after finishing the video before giving them permission to move to the game. They thought that it glitched and would close the platform and reopen it. They know how to use technology well. - The difficulty was only in registering at first and in technical issues rather than the content itself. - The barriers include technology... the lack of computer labs and proper internet connection.   **Children:**   - I faced difficulties with the email and password at first because it wouldn’t open. Then, I faced some glitches that were resolved once I used the school’s computer and then were resolved at home. - The video would also play from the start on its own and would not automatically move to the game. The page also did not open automatically. - The food diary would delete what I wrote. The baskets game would glitch sometimes and the items wouldn’t reach the baskets. - The platform did not automatically unlock the second unit after I completed the first one. Also, the platform kept glitching whenever I rotated the screen and would sometimes take me to a different page when I entered.   **Bahrain:**  **Parents**   - There were no problems except for accessing the platform and registering, and sometimes the internet connection is weak. - There was difficulty in registering and logging into the platform. Some videos would freeze and take time to load.   **Teachers:**   - The main issues were technical, mainly logging into the electronic platform. - In the food classification activity, some buttons did not work immediately.   **Children:**   - Sometimes, navigating the platform would require the user to log in again, certain buttons would become unresponsive. - Some videos would freeze or fail to load.   **Jordan:**  **Parents:**   - The account login process should be a bit easier than this, not too many accounts and complicated steps. - We took more time in the first activity since some pictures were not clear like the bulghur and the stuffed vines leaves. - Some of my daughter’s colleagues faced the issue of unavailability of more than one electronic device or cell phone in their household, so they had limited time to access the platform since their parents could not give up the device for more than an hour.   **Teachers:**   - The issue was mainly with the registration process from the start. One of the problems was the nickname field. - As for other technical difficulties, the girls told me that the transition from a game to a game sometimes glitched. - Their opinion was that it was nice and the games were entertaining, but they found it a bit difficult because, honestly, only a small number of students managed to access the platform. - Some of the students were not able to register on the platform because they did not have access to smart phones or only had one phone for the whole family so the parents could not give it to their child. - Some students also did not have internet connection, but the majority tried as much as they can to interact with the idea. Almost three quarters of the students accessed the platform.   **Children:**   - I need help at the beginning when I wanted to open the platform because I was having difficulties in putting the password, so I asked my teacher for help. After this issue was resolved, it was easy for me to use the platform on my own. - I started using the platform last Sunday. I still have one level that won’t unlock. They tell me that I need to watch the video so the activity unlocks but it won’t even when I watch the video.   **Lebanon:**  **Parents:**   - We faced a problem with the email and password every time because I did not know how to save them on the website. After registering, everything was great. She used my phone. - We tried using 3 different devices (phones and an iPad) and the platform would always glitch in the transition from the video to the game. This happened 4 times where she couldn’t move to the game. The food diary did not also work.   **Teachers:**   - It is better to simplify the registration process and the access to the platform, maybe we can let them download the activities. - The students had some difficulties in accessing the link although I explained a lot (they couldn’t find it) and then they faced difficulties in their internet connection. They only reported difficulties related to technical issues during the registration process. - The biggest difficulty was the weak internet connection and the trouble with registration. Even though the teacher wrote the steps on the board, they still didn’t understand.   **Children:**   - The password was not working. - When I first entered there was the code and the email, so I was logging In instead of registering until the teacher explained to me how it should be done.   **1.2** **Parental and Teacher Support** – Role of teachers and parents in helping children adapt to the platform.  **Palestine:**  **Parents:**   - The fact that the school was following up and the ministry was monitoring the process, as well as the certificate idea. - The motivation came from school ... because they were following up and linking the platform to school activities. - I helped my daughter, who’s excited about using the platform. - Her teacher and older sister helped her.   **Teachers:**   - The students came back to us a lot and asked for support to enter the platform. - The help and support kept flowing till the end of the platform. - A lot of students needed my help with the technical issues. Only 5 out of 28 students were able to succeed on their own.   **Children:**   - My mom helped me twice in the rainbow activity. - My father helped me and gave me his laptop and the platform did not open on the iPad. Once I was in, my older brother would help me with some games. - I needed my mom’s help in the first and last activities, and I had trouble remembering the information in the sugar and fat activity. - My parents helped me with the registration process (email and password) after I tried on my own. I used a phone and an iPad. - My friend at school helped me open the platform when it didn’t work on my laptop. - I needed support because I had to write the email and password every time I wanted to access the platform.   **Bahrain:**  **Parents:**   - Yes, there was great support from the teachers, as they even provided us with instructions on how to log into the platform. They also maintained continuous communication with us through contact channels. - Parental involvement plays a role in motivating and encouraging the child.   **Teachers:**   - It was somewhat difficult, but with our support, especially for certain activities such as measuring the number of spoons and food portions, it became manageable. - The food classification activity required assistance, as well as the portion size activity.   **Children:**   - There was sufficient support from teachers, who even communicated with parents to assist us. - All the provided materials were beneficial. - There was support from both the teachers and parents, who assisted us during the registration and login process.   **Jordan:**  **Parents:**   - She even keeps telling me, “Mom, fix it for me so I can keep playing in my free time.” She finishes her studies and then plays. I told her, "Okay, once I figure out a way to keep you connected, you’ll be able to continue using it." She’s really happy with it. - Same goes for me and my daughter. We already had a healthy lifestyle at home and she enjoyed implementing the things she learned. I felt that she was enjoying using the platform as she was finishing one activity after the other until she completed all the levels and got her certificate. She even replayed the games and called us, including her younger brother who’s in the first grade, to play with her. - We asked for the teacher’s help and there was a lot of collaboration between us on the group until the problems were solved.   **Teachers:**   - Even for me as a teacher it was hard because it required for example 12 letters and they wanted them in both capital and small, including symbols and numbers, so their entry was frankly complicated. And till now there are girls who are still not able to enter even after one month. - I encouraged the students in class with full moral support. It was a personal communication between me and the parents, and within the classes, I motivated them to access the platform. They were able to use it alone as soon as their mother opens the link and hands them the phone. The only support provided was in technical issues or registration. - I followed up with them on group chats and sometimes on private chats.   **Children:**   - I found the food groups activity hard at first but I was able to solve it with the help of my sister. - I needed my brother’s help with the registration at first and with the glitches in the seventh activity. He tried to help me with that but the issue was not fixed. My teacher told me to keep trying. - The teacher gave us great support and started telling us that these games are informative and can teach us a lot of things. - Usually, my mom opens the platform for me and I play on my own. I also have a slow internet connection which makes the platform glitch.   **Lebanon:**  **Teachers:**   - I suggest to start in class, especially to guide them in the registration process and show them how to proceed through the levels due to the restrictions in the level of knowledge of some of the parents at home. - There are a lot of technical issues. They lose motivation, you know how kids are, they don’t have the patience to keep trying multiple times to register or play the game. If it doesn’t work once, that’s it, they just give up.   **Children:**   - My parents knew about it and said it was really great and that they love doing things like this. - My mom saw it from the beginning to the end, and my younger brother was watching with me. - My mom was next to me watching, when she offered help, I told her no. |
| **2. Content Enjoyment: Storylines and Games** | Explores how students and parents perceived the interactive elements, engagement, and educational content. | **2.1** **Engagement with Games, Characters and design preference**  **Palestine:**  **Parents:**   - The concept of collecting points and competition was attractive, as well as feeling eager to move to other levels and the curiosity of seeing what’s in the next activity. - She improved on a personal level and was influenced to do things that are usually for older, like cooking on her own. - The characters are really eye catching. - The games are fun and some kind of competition was created among students on their WhatsApp group. - The platform was unique and motivating because of the way it teaches the kids through educational games. The transition from one level to another is also attractive. - The presence of cartoons and games (especially the baskets game) was fun, and she liked when she got the certificate.   **Teachers:**   - The students played the games with a competitive side because of the points and the rankings, so they wanted to see who can collect more points, and they competed among each other. - Overall, the platform is nice, fun, and beneficial. - The healthy kids program focuses on healthy food choices, breakfast and snacks whereas the things taught in the curriculum are given during one session, which makes the platform practical, reinforces the information and focuses on promoting healthy habits more than the curriculum does.   **Children:**   - I liked the characters because they are cartoon characters, funny and entertaining. The information they gave us was easy. - I would play again and would make my cousins play with me so they can cut down on the extras and eat more fruits. I did not need a motivator because I had fun on the platform. - I would recommend it to younger age groups because it is fun and so that they stay away from unhealthy foods. The platform itself is nice and encourages us to play. - The cartoons were nice and beneficial. I understood the information and was influenced. - Water is the best drink… I liked the video and the game. It taught me the importance of staying hydrated. - The platform encouraged me to organize my meals and make my food colorful.   **Bahrain:**  **Parents:**   - The platform's use of colors and characters, as well as the incorporation of videos enriched with interactive materials play a role in motivating and encouraging the child. - I personally like a healthy lifestyle, and the platform helped me reinforce this idea. - A useful platform that focuses on students' interests, changes their concepts and approaches to nutrition. At this stage, they need awareness. - The platform in general helped the children find healthier alternatives for breakfast, and an actual implementation of the things they learned took place, even if the parents did not tackle the issue of healthy eating before. - The program should be continuous, with different levels, and the addition of some competition activities.   **Teachers:**   - The students were happy with this experience and enjoyed the activities a lot because they were entertaining and allowed them to disconnect from electronic games. - The platform is an educational tool that incorporates play, a technique that motivates to grasp the information in an engaging way. - The vibrant colors, the characters, and even the soft music on the platform played a role in attracting children.   **Children:**   - The characters were great, and the story used was engaging for students at all levels. The information was easy to understand because of the storytelling method, between Farid and Fareed.   **Jordan:**  **Parents:**   - The content is suitable for their age group and convincing. For example, I used to tell my children all the time that chips and chocolate are bad for you, but they were more convinced that what I’m saying is true after accessing the platform. - The platform is using an approach similar to video games which is attractive to children and makes the information stick to their minds. I would definitely recommend it since it is a very informative platform that needs to be implemented on a broader scale.   **Teachers:**   - They enjoyed it because of the existing drawings, cartoons and videos. They enjoyed the fact that they can play and collect points, and that there is a competition to collect points from within the class and among the girls.   **Children:**   - I liked the game with the treasure map with sugar and fats. - I liked categorizing food items because we learn which ones are healthy and we need to include more in our diet, and which ones are unhealthy that we need to cut down on. - I liked the characters and wished I had a friend like Farid who could teach me such information.   **Lebanon:**  **Parents:**   - My daughter liked the games and the cartoons. She even refused to take a can of juice and asked for a cup of natural fruit juice.   **Teachers:**   - The whole content was equally easy and nice, and the students really enjoyed it.   **Children:**   - Their voices were funny, and they explain very well. The information was easy. - I loved the colors.   **2.2** **Challenges with Content** – Difficulty with certain lessons and lack of engagement with certain videos.  **Palestine:**  **Parents:**   - She had some difficulties with the food portions activity. - The content is great... but the food portions and tablespoons of sugar and fat were hard. - The food portions activity needed some more explanation ... and some keywords were hard and should be replaced.   **Teachers:**   - Memorizing the number of spoons of sugar and fats were a bit challenging. 5 students faced difficulties with their mental ability just like any other educational material.   **Children:**   - The information they gave us was easy, but some units included a lot of information, which made it hard for me to answer correctly when I played the game and had to rewatch the video. - The video where they were talking about fats and oils was a bit hard because they talked a lot. - Also, I had some trouble in pulling the food items into the rainbow activity. - The hardest activity was the ladder and snake game because memorizing the amount of fats and sugar was challenging. - The food portions one was hard, including the information provided.   **Jordan:**  **Parents:**   - Some of the pictures were not clear and were too small for the children to identify - I agree that the content is very suitable for their age group since my daughter was able to solve everything. - My daughter needed my help in the baskets activity to understand why some of the food items did not match with the baskets she was trying to put them in.   **Teachers:**   - The material is easy. I didn't feel that the students had any problem, on the contrary, they were having fun and talking to each other about it. The activities were not difficult and were appropriate for their age group. - To wrap it up, the activities that required the students to memorize a lot of new information were the hard ones.   **Children:**   - I suggest adding more new information to the “color your food” activity because it is too easy. - There are questions that are neither too easy nor too hard like the ones in the first. I asked my mother to help me with the questions I could not answer on my own. In case she could not help me, I would turn to my siblings. - I would play again but it would be better if you updated it and added more videos and new information that we can benefit from. My younger siblings used to watch some of the easy videos with me so they can learn from them too.   **Lebanon:**  **Teachers:**   - It is very important to work a lot on motivation first. Honestly, I’ll tell you it’s really important to motivate the students on the platform. So first, you need to convince them why and to give them something that persuades them. - At the beginning, the students were really excited and motivated. One student reported that she thought the platform was for younger kids.   **Children:**   - Easy but the videos were long. - Treasure game was not clear because I did not memorize the spoons of sugar and fat.   **Bahrain:**  **Parents:**   - The most challenging games were "food portions," and the “maze dice”.   **Children:**   - The least preferred were portion size and food classification because they required more focus. - The hardest ones were portion size and the maze activity.   **2.3** **Integration with Classroom Learning** – Teachers' perspectives on how the platform complements traditional teaching.  **Palestine:**  **Teachers:**   - Yes, I can use the platform in class where we can exchange information, have discussions and push the students who stopped playing to complete the activities. - We can use the “I am a hero” activity in the physical education session. I recommend implementing the platform at school in coordination with the lessons we teach to encourage the students and reinforce the information given. - I am interested in integrating the platform in the science class by including topics about healthy and unhealthy eating and linking it to body functions to make the information stick to their head.   **Bahrain:**  **Teachers:**   - I integrated the Ajyal Salima curriculum into the Healthy Mind in a Healthy Body project, as well as into the Family Curriculum. Also, the questions were incorporated into the lessons and linked the physical education curriculum with Ajyal Salima. - There were no obstacles in delivering the content; on the contrary, the content aligned well with the curriculum, was enriched with educational videos and activities, and brochures were also created. - The program was discussed and implemented in physical education classes, where students participated in preparing a healthy group breakfast. Ajyal Salima program was also incorporated into the school radio through health awareness messages.   **Jordan:**  **Teachers:**   - Using the platform will help me with teaching. If there are things that can benefit me in the educational activity, like videos for example, this will add support to the educational material especially in the midst of the technological burst going on. Developing and using technology methods can be of great help. - Integrating the platform with my lessons was very useful and more fun for the students.   **Parents:**   - Honestly, I want to say something. As a platform, and for students to learn from the start what to eat, maybe this could become a practical application—not just at home, but even inside the classroom. There could be a specific activity that we do. I know this isn't their specialty, but I’m saying this as a recommendation for the future. Maybe there could be a dedicated lesson once a week where students actually learn how to apply it.   **Children:**   - I would’ve preferred if our teachers were the one explaining the information to us and then we would play the games after.   **Lebanon:**  **Teachers:**   - Sure, especially when explaining the food pyramid I could use some of the games as a supplemental teaching tool. - I can use it. I liked the videos. We can show the videos to the students, we can play the game ourselves. Because we’re all set up—you know, the screen and everything are available in the classrooms. - I can benefit a lot from this platform in the classrooms. It's much easier than how we used to do it before—printing posters, bringing materials, and you know, with the current economic crisis, this feels like a more practical solution. |
| **3. Changes in Children’s Habits** | Examines how the digital platform influenced children's nutrition and physical activity habits. | **3.1 Nutritional Behavior Changes and Knowledge-action gap**  **Palestine:**  **Parents:**   - I noticed that she cut down on sweets and packaged noodles and started shifting to healthy foods. She also cut down on carbonated beverages and became more committed to brushing her teeth regularly. - She started eating more fruits and vegetables and made all of us cut down on unhealthy foods. She started purchasing yogurt and low calories stuff from the supermarket. - My daughter’s energy level increases, she cut down on buying junk foods, replaced chips with popcorn, and started eating more fruits and vegetables. - My daughter was surprised with the amount of sugar present in sodas and other food items, so she started paying more attention to what she eats. - Did not see any changes in my daughter's behavior, she likes eating sweets. What changed is that I am no longer getting takeaway meals, and she started asking for salads. - Her knowledge increased but her behavior did not change. - Nothing changed.   **Teachers:**   - I noticed an increase in their consumption of healthy foods like fruits and vegetables since they focused on getting 5 servings per day. Their knowledge increased and we did a revision of the food groups. As for their food intake, they cut down a bit on the foods belonging to the extra food group. - The students started paying more attention to their breakfast during the breakfast break at school (where they distribute the students’ lunch boxes) and started bringing more fruits and vegetables with them.  They also started paying attention to the food items belonging to the “extra” category and to how to cut down on them. - They are bringing lunchboxes with them to school. Their knowledge increased but the practical implementation of what they learned did not.   **Children:**   - I did not use to like fruits and vegetables, but after learning how rich they are in vitamins, I started liking them. Even my siblings got motivated to eat more fruit and vegetables after I started doing so. - I started eating a wider variety of foods and eating 5 servings of fruit daily. I also started brushing my teeth more and cutting down on carbonated beverages. - I also started making my own breakfast. - I started paying more attention to the servings I eat per day. - I started buying a sandwich and juice instead of chocolate from the school cafeteria. I cut down on purchasing chocolate, and I buy one chips. - I started exercising more and stopped frequently buying from the school’s cafeteria. I only purchased one bag of chips. - I did not change anything.   **Bahrain:**  **Parents:**   - The videos reinforced the learning that positive changes were noticed in children, such as increasing interest in portion sizes, and choosing healthier foods.   **Teachers:**   - Students’ nutritional habits and concepts have changed. They became more consistent with having breakfast, avoided fast food, soft drinks, and consuming from the extras group. - The students noticed changes, they became more consistent with having breakfast, changing their lifestyle, and avoiding chips and soft drinks.   **Children:**   - Changes occurred in our understanding and dietary habits. We reduced fast food and soft drinks, became more consistent with healthy eating. - We changed a lot, and you can see it in our daily routine. We committed to having breakfast, eating more fruits and vegetables, avoiding soft drinks, and playing sports.   **Jordan:**  **Parents:**   - When she started following the platform and seeing how these things could affect her health, she became more aware. Now that she's in fifth grade, she even noticed changes in her skin, like breakouts. I explained to her that this could be due to excessive sugar, oils, and chips. So, she even started adding more fruit to her diet. - She started teaching her older sisters what’s healthy and what’s not. Then started to tell their cousins “You eat too much; you’re eating the wrong way.” Even though their food is fine, she insists, “No, your food is not right,” acting like a health coach.” - I’m happy to see actual lifestyle changes, especially in their food choices. A mother cannot stay in control of everything. - Now, she studies with a water bottle, drinking between 6 to 8 cups of water every day. I tell her it's a lot, but she says, "No, it's not a lot. This is what I saw on the platform." - I noticed that whenever I was setting up the table, my daughter would tell her brother that he needs to have 5 servings of fruits, since he doesn’t eat fruits and vegetables much. - When she watched the video on the platform, she started brushing her teeth every day. - Even at home, she started pointing out the healthy items in her plate, the unhealthy ones and the ones that are high in oils like fries and chips, which she cut down on. She even cut down on ice cream and starting giving advice. - Changing the children’s eating patterns can be challenging especially when the rest of their social circle keeps buying junk foods. However, they are trying to implement the tips they learned on the platform. For example, they sometimes challenge each other on who can drink more water.   **Teachers:**   - They even started implementing the things they learned from the platform at school by bringing healthy lunchboxes and showing them to me. - They started bringing healthy sandwiches and healthy snacks with them to school, and cutting down on sugar, chips, chocolate and juices. In addition, they started drinking more water and playing sports. I noticed these changes since they would come up to me during class and show me how they are drinking more water. - They would also tell me how they got jealous from each other and started implementing the same lifestyle changes. For example, they started buying healthier items from the school’s cafeteria. The students understood the material and started shifting towards a healthier lifestyle, which was also reported by some parents. Some students started eating better and building healthier habits. - No they are still buying chips and chocolate. They are aware and understand, but in terms of application they did not, not even when I gave them the food lesson. - Personally, I did not notice any changes, but I understood that they retained the information they acquired from the platform.   **Children:**   - I used to drink a lot of sodas and did not like drinking milk. After accessing the platform, I started drinking milk. Same goes for fruits and vegetables. - I started eating in moderation like Farid the rabbit taught us and cut down on unhealthy foods. - I made a healthy lunchbox that had in it fruits, vegetables and nuts. We also made a juice with bananas and strawberries because it can help us focus. - Even the school supported the platform and made the system healthier, so they stopped selling chips and chocolate. Instead, they started selling pastries and even mentioned that, in the future, they will start selling fruits and vegetables. - I prepared a diet schedule on a paper that I hung in the kitchen and that helped me know how to eat at lunch. I also used to eat a lot of food that are high in fats and sugar instead of having healthy snacks or breakfast, but after using the platform, I sticked to the diet schedule I prepared and started eating healthy foods. - I used to suffer from my teeth, but after watching the videos, I learned to cut down of candies, to eat more fruits and vegetables, and to brush my teeth first thing in the morning and before going to sleep. - I did the shoebox activity with the help of my mother and started using it to count the servings of everything I eat. - My siblings and I used to have instant noodles for breakfast. After using the platform, I stopped doing that and started having eggs for breakfast instead. I plan to keep doing that.   **Lebanon:**  **Parents:**   - My daughter started to think about cutting down on sodas but still needs more motivation.   **Teachers:**   - One of the moms told me that her kid benefited from the platform which made him decrease his consumption of fast food and increased his knowledge of healthy foods. - Many children have started telling their friends, "Don't bring soft drinks, don't bring pastries...” - No lifestyle changes were reported by the students who accessed the platform due to the lack of time and decrease in motivation.   **Children:**   - I used to have more than 2 snacks, but now I stick to 2, and I also started eating fruits. - I used to drink only one cup of water. - Breakfast—when they told us what to make for breakfast and how to prepare the sandwich, I liked it and kept making it!   **3.2** **Physical Activity Changes**  **Palestine:**  **Parents:**   - My daughter started being more physically active and became more energetic. - She started doing Zumba dances with her sister... “I am the hero” game. - She also asked for a jumping rope so she can do sports. - She wants to play more sports willingly, which felt like an obligation for her before using the platform. - She started exercising more with the family.   **Teachers:**   - During our discussions, they used to express how eager they were to participate in the physical activity class so they can exercise.   **Children:**   - I also started taking care of my health and doing more physical activity like Fareed. - I started doing more physical activity every day. - I started exercising more. - I started exercising in the morning.   **Bahrain:**  **Parents:**   - The videos reinforced the learning that positive changes were noticed in children, such as increasing interest in sports.   **Teachers:**   - The students noticed changes, particularly in their activities. They became more consistent with playing sports and changing their lifestyle.   **Children:**   - We changed a lot, and you can see it in our daily routine. We committed to playing sports. - Changes occurred in our understanding and dietary habits. We became more consistent with exercising.   **Jordan:**  **Parents:**   - My child started doing exercises in front of the TV three times per week. - The sports activity. My daughter started playing with her sister, who’s in the seventh grade, and another girl. They would open the sports activity and do it together.   **Children:**   - I started doing more physical activity since I used to be more sedentary. Watching the videos motivated me to be active and taught me that sports are extremely beneficial to our body. - The sports activity motivates us to play more sports and be active which can help us increase our energy levels and make us focus more while studying. - Before using the platform, I didn’t do any kind of sports. But after using it, I started playing sports with my siblings.   **Lebanon:**  **Children:**   - I like how Farid does sport, and I do it in the morning! - I increased my physical activity. |

| **4. Recommendations to Improve the Digital Platform** | Suggestions from parents, teachers, and students for improving usability, content, and engagement. | **4.1** **Enhancing Platform Features** **and implementation:**  **Palestine:**  **Parents:**   - I encourage the idea of sharing pictures on the platform for all users to see. - I suggest developing the platform to make it accessible for all age groups. - I suggest adding more points and making it optional, as well as creating a competition between the parents and their children through a quiz, for example. - I wish there was a practical activity in between games because I felt that the overall picture wasn’t clear. Also, the students were communicating on the group, so it would’ve been better if there was a chat section on the platform itself. I also suggest making the units longer, so the information can be delivered better. I also suggest creating characters for a specific story to make it more like different episodes in a way that each unit has its own purpose. It’s like creating a 10 episodes series, for example with a united background story by changing the way the story is told. - I suggest adding a summary of the activities at the end either through pictures, voice recording or educational tool, as well as adding an option where students can upload pictures and videos of the activities they do at home. - I suggest adding more colors and characters. - I suggest adding an option to save the game. - I suggest adding a revision section at the end. - I suggest adding a section about the prevention of diseases. - I suggest adding more videos that highlight the dangers of consuming a lot of unhealthy food as well as the health consequences. - It would’ve been also better if the implementation time was longer.   **Teachers:**   - I recommend adding more videos and lessons. - It would be better to make the food diary mandatory once they log in. - Making the games easier by making the mistakes clearer. I suggest adding a limited trial and time to do the activities so they can focus on the videos, and so that the students don’t play the game more than one time to win without watching the videos. - The implementation time happened to be during the exams period and the beginning of Ramadan so there wasn’t a lot of time... It would be better if it can be implemented at the beginning of the school year.   **Children:**   - You can add some colors and make it more vibrant. - I suggest adding more videos about the vitamins and their benefits for the parents to watch. - I suggest adding more videos so we can learn about health and how to eat our meals. - I suggest adding more games. - I suggest adding more pictures of fruits and vegetables. - I suggest adding more tips so we can benefit from them. - I wish they would give us something new to learn. - I suggest adding a few more characters to the videos and decrease the number of food items in the rainbow activity. - I suggest making the videos shorter because they were too long, as well as changing the names of the characters instead of giving them the same name.   **Bahrain:**  **Parents:**   - Changing the platform's registration method so that it is accessible to others. - The platform needs challenge-based activities between two people to increase excitement, along with (notes) to allow self-reflection. - The program should be continuous, with different levels, and the addition of some competition activities.   **Teachers:**   - Adding more characters to engage children further. - The registration process should be simplified by having a unified code for the fourth and fifth levels. - The timing was inconvenient for teachers as it coincided with the second semester, the beginning of the month of Ramadan, and school holidays.   **Children:**   - Instructions should be given before starting any activity, along with increased motivation and the options for a two-player challenge. - We also need more motivation, additional levels, more points, and increased opportunities for two-player challenges.   **Jordan:**  **Parents:**   - The students can also share their own experience on the platform itself. I suggest also creating a WhatsApp group at the beginning of the school year that includes some mothers who can supervise the progress on the platform. For example, they can do a competition during each semester where mothers can log what activities her children did each day. - I would also suggest to do a competition at school after the children have finished the activities. - I would also suggest adding a section on the platform dedicated to the takeaway messages that the children can write down after finishing the activities to see what are the things they benefited from. Another thing that can be added is a section at the end in which the child has to do a project or maybe draw a picture that depicts how they benefited from the platform. - I would suggest following up with the students even after they’ve obtained their certificates. I mean opening new levels according to the physiological changes they will go through on the long run. - Also, include for example what they should be eating to correct deficiencies in some vitamins or minerals like magnesium, iron or vitamin B12. I would also like to add that mental health is also important. At this age, girls are prone to bullying due to being overweight which can be tackled through the cartoons in the platform.   **Teachers:**   - There’s nothing that I would suggest other than making the platform public in a way that makes the access to it adapted to all age groups and that makes the registration process easier.   **Children:**   - I suggest adding a section in the platform where we can learn how to make healthy versions of foods, like chips for example. - We can choose the characters ourselves and customize their clothing, name and even we can choose the animals we like. - I finished all the activities in just two days because I was bored and the activities were fun. - I also finished all the activities in two days and I used to open it twice a day.   **Lebanon:**  **Parents:**   - It's better if they stay engaged for longer so they can benefit more, rather than playing those pointless games. - They should restrict the time during which an activity could be done by locking the levels so that children have a longer time frame during which they are able to finish all the activities so that the information can stick better to their mind.   **Teachers:**   - To motivate kids to be more involved is by offering a reward or a ranking after finishing all the levels. - In my opinion the videos and activities are neutral since there are much more sophisticated and complicated ideas online. - It would be better to implement the program earlier in the academic year because, by the end of the school year, students tend to lose motivation and energy. Additionally, we should extend the duration of the activities.   **4.3** **Parental Involvement** – Challenges and Recommendations  **Palestine:**  **Parents:**   - I saw the platform once. - I did not see any of the activities with her, her sister was usually sitting next to her. But the breakfast activity caught my attention. - I did not follow up on the time it took her. She told me when she got her certificate. - I can't remember. - I’m not familiar with the games. - I was not with my daughter when she used to play. She used to play after finishing her homework. - To be honest, I did not follow up much on the platform...but my daughter had a good impression and started shifting towards healthy eating. - The teacher followed up with her more than I did.   **Children:**   - There was a lot of information given at once, and no one helped me at home.   **Jordan:**  **Parents:**   - The platform is nice but needs follow-up and commitment from parents. For example, my child learned how to decorate her plate with fruits and vegetables and got motivated to execute the idea at home and she decided to cut down on desserts and junk food and eat more fruits and vegetables. - The content of the platform is not only suitable for the children’s age group, but to all age groups since it provides information that is even beneficial to us. It is important to start implementing nutrition education at a younger age since you can convince children to follow the tips with fun activities in an easier way compared to older age groups. - My children love the platform and are interacting well with its content. I am able to watch some of it when I am sitting next to them or if I am helping them. The activity I remember the most is the one with the food groups and servings. - My daughter and I argue over who gets to play! Hahaha. It’s suitable for all ages. - I have no idea since she did the activities with her siblings. - ... Honestly, at first, I didn’t open it with her, and even now, I don’t, but I learned what the platform's content is. I asked her why, and she told me, "Mama, I want to get ahead of the girls, I want to get ahead of my friends." - They were engaged with something useful. I mean, I don’t want them sitting on YouTube watching reels. I don’t want them playing games that don’t benefit them. Here, there was something that opened their minds, they started asking questions.   **Teachers:**   - What can maybe help is to raise awareness among parents. I mean the parents must know whether it is good or bad to pack a chocolate sandwich in their kid’s lunchbox. - They weren't much interested in it. Maybe because it is not part of the curriculum or because it happened to be implemented at the end of the school year. I didn’t even feel that their knowledge of the topics increased, they told me that the information is close to what I had already given in class.   **Children:**   - I couldn’t try any recipe because my mother doesn’t allow me to access the kitchen and did not let me try any recipe. - I did not do the shoebox activity since my mother was busy and I couldn’t do it on my own.   **Bahrain:**  **Parents:**   - To be honest, we did not help them in solving the activities because the school was the biggest supporter in solving the activities.   **Lebanon:**  **Parents:**   - No, I didn’t notice the platform, but my son went in and tried, and he started making food. But he didn’t tell me anything—I just saw him making food, but I didn’t understand why.   **Teachers:**   - Difficulties faced were mainly in the registration process, especially that parents did not help their children in the process, and it is hard for kids to register on their own since they were not filling in the information correctly. - We can do an open house for parents (for an hour) to introduce them to the platform and show them what the program is about. - I was shocked that the parents did not follow-up with their children and did not even check what they are doing on the platform.   **Children:**   - Every time I ask my mom something, she tells me, "Later, I'm busy right now. |
| --- | --- | --- |
