# Supplementary material for: Successes and challenges of an online based nutrition awareness program in 9–11-year-old children in four Arab countries: The Ajyal Salima digital platform qualitative study
Source: PLoS One. 2026 Mar 11;21(3):e0325583. doi: 10.1371/journal.pone.0325583 (PMC12978466; doi:10.1371/journal.pone.0325583)
Supplement: S3 Checklist — (DOCX) [file pone.0325583.s003.docx]

Table 1 Consolidated criteria for reporting qualitative studies (COREQ): 32-item checklist

| No Item | Guide questions / description |
| --- | --- |
| **Domain 1: Research team and reflexivity** | |
| Personal characteristics | |
| 1. Interviewer/ facilitator | Independent interviewers appointed by each country’s Health or Education Ministry conducted the focus group and interviews. |
| 1. Credentials | Co-authors include researchers with different academic affiliations including universities and health ministries. CHM, AK, DT, MB, EH and NH each have a PhD. ST has an MPH. CM and LT each have BSc |
| 1. Occupation | The research team included academic researchers, and ministry staff from participating countries for conducting regular visits. |
| 4. Gender | Females |
| 5. Experience and training | All staff members received research ethics training. The interviewers attended 3 training sessions:  (1) One on Research Ethics (2) a tutorial on how to use the Ajyal Salima microsite, and (3) on how to conduct qualitative research. |
| Relationship with participants | |
| 6. Relationship established | No relationship was established prior to study commencement. |
| 7. Participant knowledge of the interviewer  *What did the participants know about the researcher? e.g. personal goals, reasons for doing the research* | Participants knew where the researchers worked and the purpose of the research. They were provided with information about the study objectives, methods, and potential risks through consent and assent procedures. No personal goals, assumptions, or motivations of the interviewers were disclosed to participants. |
| 8. Interviewer characteristics  *What characteristics were reported about the interviewer/facilitator?*  *e.g. Bias, assumptions, reasons and interests in the research topic* | Interviewers had previous experience in qualitative research, no further reporting about interviewer’s characteristics was made. |
| **Domain 2: study design** | |
| Theoretical framework | |
| 9. Methodological orientation and Theory | Thematic content analysis was used, a method described by (Burnard, 1991). Thematic content analysis is adapted from grounded theory and was carried out using a systematic approach of immersion in data, coding, and data reduction. |
| Participant selection | |
| 10. Sampling | This qualitative study employed a purposeful, multi‑stakeholder sampling strategy to capture diverse perspectives on the implementation and impact of the Ajyal Salima program across multiple sociocultural and educational contexts. |
| 11. Method of approach | The schools were contacted through the Ministry of Education in each country. Consent forms were sent to parents. |
| 12. Sample size | Total number of participants: 270  Children: 145 participants across 21 focus groups  Parents: 98 participants across 16 focus groups  Teachers: 19 participants in 18 one-on-one interviews  Ajyal Salima staff: 8 participants  Schools: 13 schools in total (4 in Lebanon, 3 each in Bahrain, Palestine, and Jordan). |
| 13. Non-participation  *How many people refused to participate or dropped out? Reasons?* | All schools who were invited accepted to participate and none dropped outs |
| 14. Setting of data collection | Data collection took place in schools across four countries (Lebanon, Bahrain, Palestine, and Jordan). |
| 15. Presence of non-participants | No |
| 16. Description of sample | Age: Schoolchildren aged between 9 and 11 years and enrolled in grades 4 and 5.  Countries: Four Arab countries (Lebanon, Bahrain, Palestine, and Jordan).  Participants: Children with their parents, classroom teachers, and Ajyal Salima staff.  Time period: Data collection from May 2023 to May 2024.  Intervention duration: One month |
| Data collection | |
| 17. Interview guide | Focus group discussions and in-depth interviews were conducted using a set of pre-determined core questions, however they were intentionally designed to encourage open (informal) conversations, allowing participants to express their views freely. |
| 18. Repeat interviews | No interviews were repeated |
| 19. Audio/visual recording | All sessions were recorded via digital recorders to ensure that all information/feedback provided by participants was documented and to preserve the authenticity of the feedback. |
| 20. Field notes | Field notes were taken during school visits and shared with the team |
| 21. Duration | Each of the in depth-interviews and focus group lasted approximately 40 minutes. |
| 22. Data saturation | When data saturation was reached, no further data collection was pursued |
| 23. Transcripts returned | No transcripts were returned |
| **Domain 3: analysis and findings** | |
| Data analysis | |
| 24. Number of data coders | One coder  DT coded the interviews, logbooks and reports and classified the codes. The coding and description of results were verified by CHM and CM and discussed until consensus was reached |
| 25. Description of the coding tree | Quotes were inductively organized around four main themes: 1)Usability and Support using the Digital Platform (with sub-themes on technical challenges and parental / teacher support); 2) Content Enjoyment of Story Lines and Games (with sub-themes on engagement, challenges with content, and integration with classroom teaching);3) Changes in Children’s habits (with sub-themes on nutritional behavior changes, and physical activity changes); 4) Recommendations to Improve the Digital Platform (with sub-themes on enhancing platform features and parental involvement). A mind map illustrating the main themes and respective subthemes was provided as a supplementary document (S1 Figure). |
| 26. Derivation of themes | Themes were derived from the data. Quotes were inductively organized around four main themes. |
| 27. Software | NVivo software and established qualitative analysis procedures were used for data review. |
| 28. Participant checking  *Did participants provide feedback on the findings?* | Participant did not provide feedback on the findings |
| 29. Quotations presented | Participant major quotations were presented in the results section to illustrate themes and sub-themes. Quotes were identified by participant type (parent, teacher, student or staff); country (Lebanon, Bahrain, Palestine, and Jordan). Additional quotes are presented in a supplementary table |
| 30. Data and findings consistent | Findings were well-supported by the quotations. Themes are clearly illustrated with multiple quotes from different participant types and countries. |
| 31. Clarity of major themes | Four major themes were clearly presented: 1) Usability and Support using the Digital Platform; 2) Content Enjoyment of Story Lines and Games; 3) Changes in Children’s Habits; 4) Recommendations to Improve the Digital Platform |
| 32. Clarity of minor themes | Sub-theme discussions within each major theme and presents diverse perspectives. For example, for theme 3: challenges in children’s habits, the paper discusses both positive changes and knowledge-action gap, whereby some participants reported increased nutrition knowledge with no behavioral changes. Differences between countries were also highlighted. |
