# Supplementary material for: Successes and challenges of an online based nutrition awareness program in 9–11-year-old children in four Arab countries: The Ajyal Salima digital platform qualitative study
Source: PLoS One. 2026 Mar 11;21(3):e0325583. doi: 10.1371/journal.pone.0325583 (PMC12978466; doi:10.1371/journal.pone.0325583)
Supplement: Attachment — Submitted filename: PONE-D-25-25805_Aug 12 2025.pdf [file pone.0325583.s004.pdf]

Successes and Challenges of an online based nutrition awareness program in 9–11-year-old children In Four Arab Countries: The Ajyal Salima digital platform Qualitative study

The manuscript addresses an important and timely topic, particularly in assessing the feasibility of digital platforms in education—a crucial area as the world increasingly embraces digital transformation. The authors have done a great job in exploring the feasibility of a digital approach, which holds potential to shift traditional paper-based education toward more engaging digital methods, especially for children who need to learn through play and games.

To further strengthen the manuscript, the manuscript would benefit from clearer methodological detail, more concise and organized results, and a deeper interpretation of findings in the discussion. I encourage the authors to carefully revise these areas to enhance the clarity and impact of the work.

Below are comments for the authors consideration to improve the manuscript.

Title:

I suggest removing "Qualitative Study" from the title to improve clarity and impact. If the authors wish to keep it, then additional details on qualitative rigor, including trustworthiness, should be included in the manuscript, and frameworks such as COREQ (Consolidated Criteria for Reporting Qualitative Research) should be provided as a supplementary document.

Abstract:

Line 2-3: 'Digital technologies are increasingly influencing children's lives, with many seeking digital platforms for nutritional education.' This sentence is not clear, does many refer to parents or children? It would be good to start an abstract with a strong sentence.

Please include details such as the size and number of the FGDs, type of interview IDI or KII , how many themes? What software was used for the analysis? Clarify also if the FGDs were done separately for parents and children? This information is necessary to be included in the abstract.

Materials and Methods:

Study participants

Which specific interview technique was used? As mentioned in the abstract, provide details on the composition and size of the FGDs and the total number of interviews. What criteria was used for the school selection, and how were the students identified and recruited? How many were reached and actually interviewed?

Intervention

(Lines 106–116): The description of the intervention lacks details for readers to fully understand the intervention. Consider listing the 10 modules rather than only mentioning the concepts covered, which will give readers an idea of what was implemented. Also include details of the timing within the academic year, platform format (app or web-based), duration per module, sequence of modules, and length of the intervention period. Elaborate on the role of parents and teachers beyond account setup (e.g., supervision, reinforcement, technical support).

Data collection

Lines 122 –126 – The timeline is unclear. It's mentioned that the intervention lasted around a month, but many students completed the modules in a week or less. Were FGDs and interviews conducted immediately after each participant finished or only after the month-long intervention was completed? Clarifying this is important for understanding recall bias.

Sample size and Sampling: Provide a rationale for the sample size (this hasn't been mentioned anywhere) and clarify the sampling strategy, including inclusion and exclusion criteria.

Lines 130 – 133 – The phrase *"two FGDs per category"* is unclear. Please clarify what "category" refers to (e.g., country, grade level, or participant type). Were FGDs conducted separately for each subgroup, or were participants mixed? Indicate whether the FGDs were conducted per country, per class, or across the whole sample, and provide the total number of FGDs held. Also specify session duration (it appears interviews lasted approximately 40 minutes, but was this the same for FGDs?) and whether there were any notable differences in the discussions between grade 4 and grade 5 students.

Lines 134 – 136- Please state how many teachers were interviewed in total and how many schools they represented. Similarly, report the total number of students and parents who participated in

FGDs. This will give readers a clear sense of the sample size and coverage across participating schools.

Confirm whether interview guides and procedures were consistent across countries.

Data transcription: it's not clear what was used for recording? The phrase ‘auditory means’ is not clear. Who was involved in the translation of the transcripts to English?

Result

Lines 176 – 177: This information regarding the number of schools and data collection sites is more appropriate for the methods section rather than the results. Please move it accordingly.

Table 1: In Row 5 under “Number of Teachers Interviews,” the entry “3 (4)” seems inconsistent or possibly a typo. Please verify the correct number of interviews and participants.

I suggest adding a table before Table 1 to present the participant profile (children, parents, teachers) in a separate table that summarizes their demographics and distribution for all countries.

Lines 202 – 207 It is unclear if the quotes listed here are from a single participant or multiple participants. Please clarify and specify the participant type (child, parent, teacher) for each quote to aid transparency and context.

Lines 208 – 215 The Results mention that teachers’ focus groups confirmed technical concerns. However, the Methods section states that only interviews were conducted with teachers (not focus groups). Please check this inconsistency.

Quotations throughout the Results: It is recommended to indicate the participant type (e.g., Parent, Teacher, Child) and their country at the end of each quote, for example: “Quote text.” (Parent, Palestine); “Quote text.” (Teacher, Jordan); “Quote text.” (Student, Lebanon).

Overall, the result section is quite long and too much information is presented, and the authors can reduce this section by reducing the number of quotes, focusing on the key findings, and avoiding unnecessary details such as narrating procedures.

Discussion

Is too long and needs to be reduced, and it's good to consider restructuring the discussion so it follows this logical flow:

Overall – in the first paragraph, a summary of the findings needs to be written, and this is a brief restatement of key findings (not repeating the results). Followed by an interpretation of the findings in light of your data, supported by literature, and the final paragraph should be more of the implications for practice and policy. Explain what the findings mean, why they matter, how they relate to existing literature, implications, limitations, and possible future directions.

Some of the paragraphs narrate a general background which not related to the result, and these need to be condensed to make the discussion clear and readable.

Strengths and weaknesses

The Strengths and Limitations section mentions practical points but lacks critical reflection on the study's methodological strengths and weaknesses. It should better address issues like sampling, data quality, potential biases, and translation processes to enhance transparency and rigor.

Conclusion – needs to be condensed to only highlight the key conclusion based on the study findings and add recommendations.

Overall, this manuscript addresses a valuable and timely topic with promising potential for digital nutrition education. With focused revisions to clarify methodology, streamline results, and deepen interpretation, the manuscript will significantly strengthen its contribution to the field.
