# Supplementary material for: Successes and challenges of an online based nutrition awareness program in 9–11-year-old children in four Arab countries: The Ajyal Salima digital platform qualitative study
Source: PLoS One. 2026 Mar 11;21(3):e0325583. doi: 10.1371/journal.pone.0325583 (PMC12978466; doi:10.1371/journal.pone.0325583)
Supplement: Attachment — Submitted filename: reviewer comments.docx [file pone.0325583.s005.docx]

Thank you for the opportunity to review the manuscript; “*Successes and Challenges of an online based nutrition awareness program in 9–11-year-old children In Four Arab Countries: The Ajyal Salima digital platform Qualitative study*”. The paper has the potential to add detailed insights for adapting and implementing a digital version of the Ajyal Salima program. Below are my comments to help strengthen clarity, completeness of reporting, and interpretability of findings.

Major comment

1. Use a qualitative reporting guideline (e.g., COREQ^1^).

- Please select and apply an established reporting guideline for qualitative studies. The COREQ (Consolidated Criteria for Reporting Qualitative Research) checklist would be appropriate for interview and focus group–based qualitative work. Using COREQ (or another suitable guideline) will help ensure that key details are transparently reported (e.g., researcher characteristics and reflexivity, sampling strategy, data collection procedures, analysis process, and techniques to support trustworthiness).

Specific comments

1. Provide a concise summary of the original Ajyal Salima intervention components.

- Page 6, line 106 (and/or Introduction, page 4, line 62): Please include a brief, structured description of the core components of the original (non-digital) Ajyal Salima intervention, so readers can understand what is being digitized and what “fidelity” means in this context.
  - Also consider a short table/box summarizing details of both versions of the intervention components (e.g., specify target population, setting, key activities/materials, delivery agents, dose/frequency, and hypothesized mechanisms).

2. Map the original and digital intervention components to a behavioral theory.

- To strengthen the conceptual framing of the adaptation process, please consider mapping the intervention components of both the original and the digital Ajyal Salima using a behavioral change framework. For example, the Behavior Change Wheel (BCW^2^), including intervention functions (e.g., education, training, persuasion, enablement, environmental restructuring, modeling).
- This mapping can clarify what functions are preserved, modified, added, or potentially lost in the digitization process, and it can help the reader understand how the platform is expected to influence behaviors.

3. Include Ajyal Salima staff perspectives in the thematic structure to enable comparison.

- Page 26, line 566: please consider presenting staff feedback within the same subthemes used for children, parents, and teachers. This would improve triangulation and allow readers to compare insights across participant groups within the same topics.
- The Results and Discussion sections would benefit from tighter organization and more concise writing. Where possible:
  - Group overlapping ideas and avoid repeating participant quotes that make the same point.

4. Clarify next steps for evaluation and scale-up of the digital platform.

- The Discussion would be strengthened by explicitly outlining the next steps for evaluating and/or scaling up the digital Ajyal Salima platform. You may consider using the MRC guidance for developing and evaluating complex interventions^3^ to structure this section. For example, describing what should happen next in terms of: further development/refinement of the intervention, feasibility/piloting (including engagement metrics and implementation outcomes), effectiveness evaluation (and potential study designs), and implementation and scale-up considerations.

References

1	Tong, A., Sainsbury, P. & Craig, J. Consolidated criteria for reporting qualitative research (COREQ): a 32-item checklist for interviews and focus groups. *International journal for quality in health care : journal of the International Society for Quality in Health Care* **19**, 349-357, doi:10.1093/intqhc/mzm042 (2007).

2	Michie, S., van Stralen, M. M. & West, R. The behaviour change wheel: a new method for characterising and designing behaviour change interventions. *Implementation science : IS* **6**, 42, doi:10.1186/1748-5908-6-42 (2011).

3	Skivington, K. *et al.* A new framework for developing and evaluating complex interventions: update of Medical Research Council guidance. *BMJ (Clinical research ed.)* **374**, n2061, doi:10.1136/bmj.n2061 (2021).
